# Targeting the IGF-Axis in Cultured Pediatric High-Grade Glioma Cells Inhibits Cell Cycle Progression and Survival

**DOI:** 10.3390/ph16020297

**Published:** 2023-02-14

**Authors:** Yinhsuan Michely Chen, Matthew Leibovitch, Michele Zeinieh, Nada Jabado, Pnina Brodt

**Affiliations:** 1 Department of Medicine, Division of Experimental Medicine, McGill University, Montreal, QC H4A 3J1, Canada; 2 The Research Institute of the McGill University Health Center, 1001 Décarie Blvd, Glen Site, Montreal, QC H4A 3J1, Canada; 3 Department of Paediatrics and Human Genetics, McGill University, Montreal, QC H3A 0C7, Canada; 4 Department of Surgery, McGill University, Montreal, QC H3G 1A4, Canada; 5 Department of Oncology, McGill University, Montreal, QC H4A 3T2, Canada

**Keywords:** pediatric glioma, IGF signaling, the IGF-Trap, nuclear translocation

## Abstract

Pediatric high-grade gliomas (pHGG) accounts for approximately 8–12% of primary brain tumors in children. Prognosis is poor, with a median survival of 9–15 months. Insulin-like growth factor 1-receptor (IGF-1R) gene amplifications have been identified in high-grade gliomas and may contribute to its highly aggressive phenotype, but the effect of IGF inhibitors on pHGG is yet to be determined. In the present study, we analyzed the response of patient-derived pediatric high-grade glioma cells to a novel IGF-1R inhibitor, the IGF-Trap. Using immunohistochemistry, we found that IGF-1R was localized to both the nucleus and cell membrane in different pHGG patient-derived xenograft (PDX) lines under basal conditions. In response to ligand binding, nuclear levels of the receptor increased, and this was associated with the transcriptional upregulation of both the receptor and cyclin D1, suggesting that IGF-1R could regulate its own expression and cell cycle progression in these cells. Insulin-like growth factor-1 (IGF-1) increased the proliferation of the pHGG cells DIPG13 and SGJ2, and this could be blocked by the addition of the IGF-Trap. The IGF-Trap reduced the colony formation of these cells in an optimal growth medium and impeded the ability of IGF-1 to rescue DIPG13 cells from starvation-induced apoptosis. Collectively, these results implicate the IGF-1 axis in the regulation of cell cycle progression, cellular proliferation, and cell survival in pHGG, and identify the IGF-axis as a target and the IGF-Trap as a potential inhibitor of this axis in pHGG.

## 1. Introduction

Brain cancer is the second most common cancer and the leading cause of cancer-related death in children. Although advances have been made in the classification and treatment of this disease based on molecular features, the prognosis for high-grade gliomas (WHO Grade 3 and 4) remains poor. While the cause remains unknown, genetic mutations, including gene amplifications and deletions, increase the risk of developing brain tumors. Among them, gene alterations in receptor tyrosine kinases and other cell cycle regulators are highly prevalent in hemispheric pediatric high-grade gliomas (pHGG), while a Lys27Met substitution in histone H3 (H3K27M) is a molecular signature of diffuse midline gliomas [1]. Previous studies have shown that the insulin-like growth factor-1 receptor (IGF-1R) gene is amplified at high frequency in pediatric high-grade gliomas and is the second most frequently amplified gene in diffuse intrinsic pontine glioma (DIPG) [2,3]. Moreover, nuclear localization of IGF-1R has been associated with advanced-stage pHGG [4].

Insulin-like growth factors (IGF-1 and IGF-2) are mediators of cell growth and differentiation. IGF-1 is essential for early brain development, where it has been shown to promote neuronal proliferation and glial cell survival [5]. Its levels in the brain have been shown to decrease drastically after the perinatal period [5]. IGF-2, on the other hand, is expressed mainly in mesenchymal tissue and peaks during embryonic development [6]. IGF signaling is triggered when IGF ligands bind to the IGF-1 receptor, activating downstream signal transduction cascades including the Phosphoinositide 3-kinase/Serine threonine kinase 1 (PI3K/Akt) and extracellular signal-regulated kinase/mitogen-activated protein kinase kinase (MEK/ERK), signaling that mediate cell’s survival and proliferation. Ligand binding also triggers receptor modification via small ubiquitin-like modifier protein–1 (SUMO-1) and nuclear translocation via vesicular transport [7]. Nuclear IGF-1R can bind to the enhancer-like regions of several promoters, activating the transcription of various genes including its own and that of cyclin D1. Thus, receptor activation can regulate cell cycle progression and cell proliferation, which are critical for tumor progression and metastasis, via several different pathways [8].

The IGF-Trap is a soluble form of hIGF-1R consisting of the entire extracellular domain of the receptor-fused Fc domain of hIgG_1_. It inhibits IGF-signaling by binding to IGF-1 and IGF-2 in the circulation, reducing their bioavailability and impeding activation of the cognate receptor. The bioengineering and optimization of this novel IGF-signaling inhibitor were described in detail previously [9,10]. In pre-clinical studies, the IGF-Trap could inhibit the growth of several very aggressive tumors, including triple negative breast cancer cells and metastatic colon and lung carcinoma cells [9,10,11]. A new, highly effective variant of the IGF-Trap was recently produced with increased anti-tumorigenic potency [10]. Its effect on glioma growth has not yet been evaluated.

There is a compelling body of evidence implicating the IGF axis in glioma progression. Studies based on pre-clinical glioma models, including our own, have identified this receptor as a therapeutic target in this disease [12,13]. However, there is presently a relative paucity of animal models for pHGG, and there is scant information on its role in this highly aggressive disease. A recent study revealed a significant association between high/moderate IGF-1R expression and poor survival in pHGG and found that IGF-1R increased the radio-resistance of pHGG cells, identifying it as a potential target for increased therapeutic efficacy [14]. The objective of this study was to investigate IGF signaling in clinically relevant pHGG models and assess the therapeutic potential of the IGF-Trap in the treatment of these cancers.

## 2. Results

### 2.1. The IGF-1R Is Expressed in the pHGG Cells Selected, and Its Signaling Can Be Blocked by the IGF-Trap

**Figure 1:**
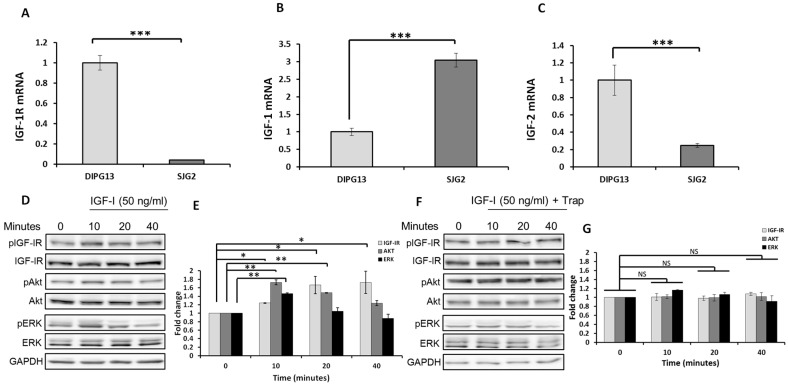
**IGF-IR activates MEK/ERK and PI3K signaling in DIPG13 cells.** Shown in (**A**–**C**) are results of qPCR analysis performed on RNA extracted from cells cultured in the respective optimal media, as described in the methods. Results are based on three analyses and are expressed relative to mRNA levels in DIPG13 cells that were assigned a value of 1. Shown in (**D**) are representative results of Western blotting performed following culture of DIPG13 cells in growth-supplements-depleted medium overnight, followed by incubation of the cells with (or without) 50 ng/mL IGF-1 for the duration indicated. Shown in (**F**) are representative results of Western blotting performed on lysates of DIPG13 cells cultured as in (**D**) in the presence of IGF-Trap that was added at a molar ratio of 2:1 to IGF-1. Shown in the bar graphs (**E**,**G**) are the means and SE (n = 4 for each set of experiments. Experiments performed separately) expressed as fold change in activation levels relative to basal levels (time 0) that were assigned a value of 1. * *p* ≤ 0.05, ** *p* ≤ 0.01, *** *p* ≤ 0.001, NS—not significant. pAKT—phosphorylated (activated) AKT, pERK—phosphorylated (activated) ERK.

The role of the IGF axis in pHGG progression is not yet fully understood. Our aim was to evaluate IGF signaling in clinically relevant pHGG models. We began by evaluating the response to IGF-1 in several pHGG patient-derived xenograft (PDX) lines. We first compared the expression levels of IGF-1R and its ligands in a library of pHGG PDXs RNA-sequencing data [15] and selected DIPG13 for further study based on a transcriptomic profile of high IGF-1R and mid/low IGF-ligand expression. These cells have an undifferentiated phenotype and harbor the characteristic H3.3 K27M substitution frequently identified in midline tumors [1,16]. They are therefore representative of traits characteristic of the clinical disease. In addition, we also used the SJG2 cells derived from a grade IV infant-type hemispheric glioma expressing wild-type H3 and harboring a Met fusion gene. These cells are representative of the H3/IDH WT subgroup of pHGG that consists largely of hemispheric tumors [17,18]. We measured the mRNA levels of IGF-1R and its ligands IGF-1 and IGF-2 in DIPG13 and SJG2 cells using qPCR and confirmed the expression of the receptor and both ligands in both cells; although, IGF-1R and IGF-2 mRNA levels were 25 fold and 4 fold higher, respectively, in the DIPG13 cells, while the SJG2 cells expressed 3 fold higher levels of IGF-1 mRNA (Figure 1A–C).

Having confirmed high IGF-1R expression levels in the DIPG13 cells, we next analyzed IGF-1R-initiated signaling in these cells. We found that, following stimulation with 50 ng/mL IGF-1 (the concentration pre-determined as optimal for these cells), IGF-1R activation could be observed within 10 min, and this triggered ERK and PI3K/Akt signaling (Figure 1D).

We previously described the bioengineering, characterization and optimization of an IGF-1R signaling inhibitor—the IGF-Trap. The IGF-Trap, a soluble form of IGF-IR, consists of the entire extracellular domain of the IGF-1 receptor fused to the Fc domain of hIgG_1_. It binds the ligands IGF-1 and IGF-2 (but not insulin) with high affinity, reducing their bioavailability to the cognate receptor and inhibiting receptor activation and signaling [9,10]. When the effect of the IGF-Trap on receptor activation in DIPG13 cells was analyzed, we found that ligand-induced IGF-1R activation and downstream signaling were significantly inhibited in the presence of the IGF-Trap (Figure 1F).

Intriguingly, we obtained different results for SJG2 cells. While receptor activation in the presence of exogenous IGF-1 was observed in these cells, no increase in Akt or ERK phosphorylation were observed, and in fact, a reduction in their activation levels was evident over time (Supplementary Figure S1A,B). This may be due to the constitutively high Akt and ERK activation levels in these cells resulting from autocrine IGF-1R activation by endogenously produced IGF-1 or deregulated Met signaling [19,20,21,22], suggesting that IGF-signaling in pHGG cells is ultimately determined by the cell context.

### 2.2. In Response to Ligand Binding, the IGF-1R Is also Transported to the Nucleus in pHGG Cells

**Figure 2:**
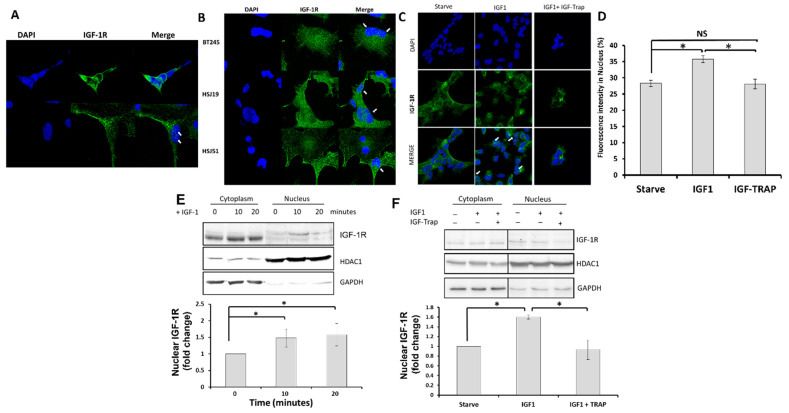
**The IGF-1R is transported to the nucleus in response to ligand binding, and this is blocked by the IGF-Trap.** The pHGG cells were seeded on laminin-coated coverslips and permeabilized (or not) with 0.1% Triton X-100, and cellular IGF-1R distribution was analyzed by immunocytochemistry (ICC). Shown in (**A**) are images of DIPG13 cells immunostained with antibodies to IGF-1R without (Top) or with (bottom) prior permeabilization. Punctate IGF-1R clusters in the DAPI-stained nuclei (blue) are indicated with white arrowheads. Nuclear IGF-1R was also observed in several other grade IV pHGGs, as shown in (**B**). To determine whether IGF-1 could increase nuclear translocation of IGF-1R, DIPG13 cells in minimal medium were treated with 50 ng/mL of IGF-1 for 20 min, and the subcellular distribution was analyzed. Shown in (**C**,**D**) are results of ICC where IGF-1R is indicated by arrowheads in (**C**), and mean fluorescence intensity measured in the nuclei based on a total of 5–9 images per condition is shown in the bar graph (**D**). Shown in (**E**,**F**) are results of Western blotting performed on subcellular DIPG13 fractions isolated following treatment of the cells with IGF-1 only (**E**) and in the presence (or absence) of IGF-Trap (**F**). GAPDH and HDAC1 were used as markers of enrichment and as loading controls for the cytosolic and nuclear fractions, respectively (**E**,**F**). Shown in the bar graphs (bottom) are results of densitometry expressed as fold change in nuclear IGF-1R levels relative to levels at time 0 that were assigned a value of 1 (n = 3). * *p* ≤ 0.05, NS—not significant.

Nuclear transport of IGF-1R has been identified as an adverse prognostic factor in different cancers and is observed in pHGG [4]. We sought to determine whether ligand binding also induced the nuclear transport of IGF-1R in DIPG13 cells. We first analyzed the subcellular distribution of the receptor under basal conditions using immunocytochemistry (ICC) and observed both membranous and nuclear IGF-1R in these cells (Figure 2A). This was also seen in other pHGG PDX-derived cells, including lines BT2545, HSJ19 and HSJ51 [16] (Figure 2B), suggesting that the nuclear translocation of this receptor was broadly relevant to pHGG pathology.

#### Ligand Binding Increases Nuclear Levels of IGF-1R, and This Is Blocked by the IGF-Trap

To assess how the nuclear translocation of IGF-1R is affected by ligand binding, we analyzed the cells following treatment with IGF-1 using ICC and subcellular fractionation. In DIPG13 cells treated with 50 ng/mL IGF-1 in minimal medium, ICC revealed increased nuclear IGF-1R levels, as compared to controls (Figure 2C,D). This was confirmed by the subcellular fractionation of the cells, followed by Western blotting, where an increase in IGF-1R levels was observed in the nuclear proteins’ enriched fraction within 10 min of IGF-1 stimulation (Figure 2E,F), suggesting that ligand-mediated IGF-1R activation increased the nuclear transport of the receptor in these cells. The increase in nuclear levels was blocked in the presence of the IGF-Trap (Figure 2C,D,F), confirming that receptor activation was essential to increased nuclear transport. Of note, IGF-1R was shown to undergo SUMOylation upon activation and nuclear translocation [7], and this could have resulted in a slight shift in its molecular weight (SUMO-1 protein is predicted to be 11.5 kDa) [23] (see Figure 2E).

### 2.3. Nuclear IGF-1R Activates Gene Transcription in DIPG13 Cells

**Figure 3:**
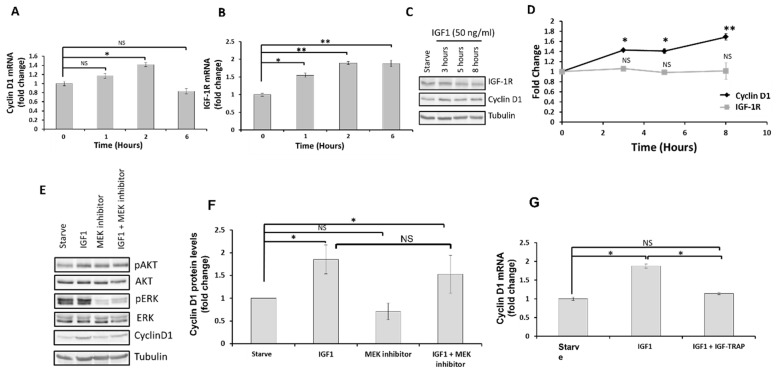
**Ligand-induced transcriptional upregulation of cyclin D1 and IGF-1R in DIPG13 cells is MEK/ERK-signaling independent.** DIPG13 cells were starved overnight and stimulated with 50 ng/mL IGF-I. RNA was collected at the indicated intervals and analyzed by qPCR. Shown in (**A**,**B**) are qPCR results for Cyclin D1 and IGF-1R mRNA, respectively. In (**C**) are representative results of Western blotting performed on these cells showing increased Cyclin D1 protein levels in the presence of IGF-1, and in the graph of (**D**) are results of densitometry expressed as fold change in Cyclin D1 and IGF-1R levels relative to levels at time 0 that were assigned a value of 1 (n = 3). Shown in (**E**) is a representative result of Western blotting performed following a 16 h stimulation of the cells with 50 ng/mL IGF-I with or without 20 μM of the MEK inhibitor PD98059; in the bar graph (**F**) are results of Western blotting expressed as means of fold change (±SE) relative to control levels (serum free conditions) that were assigned a value of 1 (n = 3). Shown in (**G**) are results of qPCR performed on cells stimulated with 50 ng/mL IGF-I in the presence or absence of the IGF-Trap. * *p* ≤ 0.05, ** *p* ≤ 0.01, NS—not significant.

Nuclear IGF-1R was previously reported to auto-regulate its own transcription as well as increase the transcription of other genes, including cyclin D1 [24]. We tested the effect of ligand-induced IGF-1R activation on IGF-1R and Cyclin D1 mRNA levels using quantitative PCR and found that both genes were upregulated in the DIPG13 cells following IGF-1 stimulation (Figure 3A,B). Western blotting confirmed an increased cyclin D1 production in these cells, although we did not detect a measurable increase in IGF-1R protein levels in the total cell lysates (Figure 3C,D), possibly due to the increased presence of IGF-1R in the nuclear fraction.

#### Increased Cyclin D1 and IGF-1R Expression Is ERK-Activation Independent

To ascertain that an increased cyclin D1 and IGF-IR expression is independent of the MEK/ERK signaling pathway activation, we treated IGF-1-stimulated DIPG13 cells with the MEK inhibitor PD98059. We confirmed the blockade of ERK signaling in the presence of this inhibitor (Figure 3E,F). However, in the presence of IGF-1, the increase in cyclin D1 expression, as assessed by qPCR and Western blotting, was not significantly altered, suggesting that it was likely due to direct regulation by nuclear IGF-1R (Figure 3E,F). As expected, the increase in cyclin D1 expression levels was also blocked in the presence of the IGF-Trap (Figure 3G).

### 2.4. Increased Cyclin D1 Expression in Response to IGF-1-Induced Nuclear Transport Is Cell Specific

**Figure 4:**
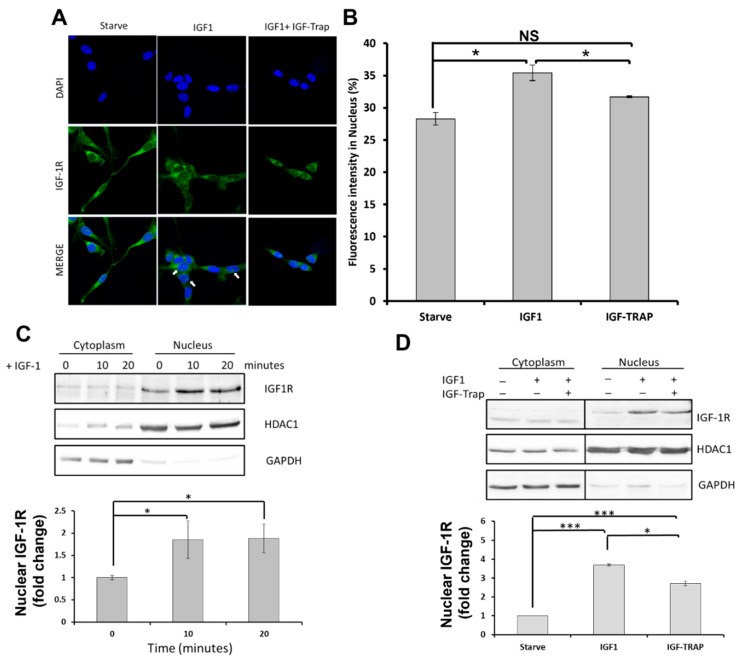
**IGF-1 triggers nuclear translocation in SJG2 cells, and this is blocked by the IGF-Trap.** SJG2 cells were seeded on coverslips, permeabilized with 0.1% Triton X-100 and cellular IGF-1R distribution was analyzed by immunocytochemistry (ICC). Shown in (**A**) are images of SJG2 permeabilized cells immunostained with antibodies to IGF-1R. Punctate IGF-1R clusters in the DAPI-stained nuclei (blue) are indicated with white arrowheads and quantification shown in (**B**). Western blots shown in (**C**) were performed on subcellular fractions obtained after overnight serum starvation, followed by addition of 10 ng/mL IGF-1 for 20 min (**C**) and in the absence or presence of IGF-Trap added at 2:1 ratio with IGF-1 (**D**). Results of densitometry are expressed as fold change in the indicated protein levels relative to levels at time 0 that were assigned a value of 1 (n = 3). * *p* ≤ 0.05, *** *p* ≤ 0.001, NS—not significant.

We also analyzed the effect of IGF-1 on nuclear levels of IGF-1R in SJG2 cells and the changes in IGF-1R and Cyclin D1 expression in these cells. We found that IGF-1 treatment also increased the level of IGF-1R in the nuclear proteins-enriched fraction of SJG2 cells, and this was blocked by the IGF-Trap (Figure 4A–D). However, in these cells, we could not detect a significant increase in cyclin D1 expression levels for up to 6 h post stimulation, and although IGF-1R mRNA levels did increased by 50%, this was not reflected in a significant increase in protein levels as was observed with DIPG13 cells (Supplementary Figure S2A–D), indicating that the transcriptional effects of nuclear IGF-1R are cell specific.

### 2.5. IGF-1R Signaling Promotes Survival and Proliferation in Pediatric High-Grade Glioma Cells

Having observed IGF-1R signaling and nuclear transport in the pHGG cells, we next evaluated the consequences of receptor activation on cellular functions known to be regulated by IGF-1R and the effect of IGF-1R signaling blockade by the IGF-Trap.

#### 2.5.1. Increased Proliferation of pHGG in the Presence of IGF-1 Is Blocked by the IGF-Trap

**Figure 5:**
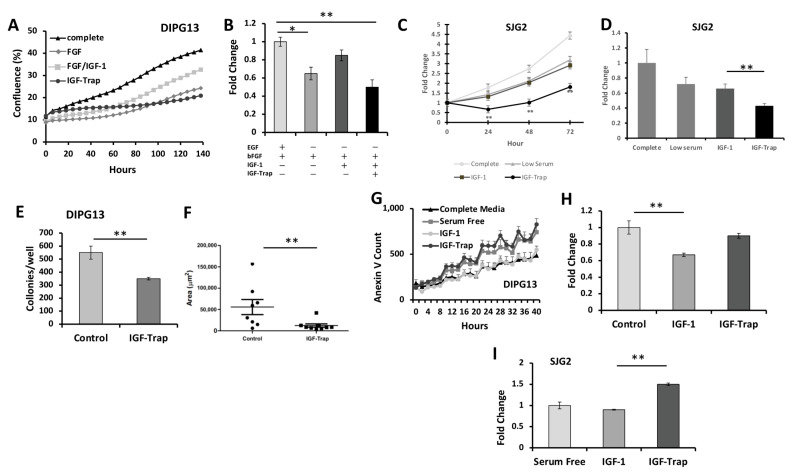
**The IGF-Trap inhibits tumor cell survival and the growth-promoting effects of IGF-1**. Cells were seeded in 24-well plates and cultured in minimal media containing (or not) 50 (DIPG13) or 10 (SJG2) ng/mL IGF-1. Shown in (**A**) are results obtained with DIPG13 cells using the Incucyte system, and in the bar graph (**B**) the results are expressed as means (±SE) relative to cells culture in optimal defined medium that were assigned a value of 1. Shown in (**C**) are results of an MTT assay performed with SJG2 cells that were cultured in serum-low medium with or without IGF-1 and IGF-Trap for the indicated duration, and in (**D**) are results of an Incucyte assay performed with SJG2 cells expressed as a ratio to values at time 0. These experiments were performed in triplicate. The effect of IGF-1 on tumor cell clonogenicity (**E**–**G**) was measured following seeding of DIPG13 cells in 6-well plates at a density of 400 cells/plate in optimal growth medium also containing 50 ng/mL IGF-1 and in the presence or absence of IGF-Trap for 15 days. Shown in (**E**) are the number of colonies counted per plate, expressed as means (±SE), of three plates, and in (**F**) is the size of individual colonies as measured using an ocular grid. Apoptosis was analyzed in DIPG13 and SJG2 cells that were cultured in growth factor and serum-depleted medium, respectively, supplemented (or not) with IGF-1 and in the presence or absence of IGF-Trap. The Incucyte system was used to monitor incorporation of fluorescently labelled Annexin V in real time. Shown in (**G**) are representative Incucyte-generated plots for DIPG13 cells (n = 3), and in the bar graph (**H**) are the results expressed as means (±SE) relative to cells in basal conditions that were assigned a value of 1. Shown in (**I**) are the results obtained for SJG2 cells expressed as mean fold change relative to cells in full medium (n = 3). * *p* < 0.05, ** *p* < 0.01.

Proliferation was measured in real time using the Incucyte system. DIPG13 cells were incubated for 96 h with or without 50 ng/mL IGF-1 and in the absence or presence of the IGF-Trap, which was added at molar ratios of 1:1 or 2:1 (IGF-Trap:IGF-1). We observed increased proliferation in the presence of IGF-1, and this was inhibited by the IGF-Trap (Figure 5A,B). We used the MTT assay to measure proliferation of SJG2 cells in the presence of 10 ng/mL IGF-1 (Figure 5C,D). While IGF-1 did not significantly increase the proliferation of SGJ2 cells in the presence of low FBS concentrations, the proliferation was still reduced in the presence of the IGF-Trap (Figure 5C,D), suggesting that it may have inhibited autocrine IGF signaling.

#### 2.5.2. IGF-Trap Can Reduce Colony Formation by DIPG13 Cells

IGF-1R is known to regulate tumor cell clonogenicity [25]. We tested the effect of the IGF-Trap on colony formation by DIPG13 cells seeded in 6-well plates at a density of 400 cells/well in an optimal growth medium supplemented with 50 ng/mL of IGF-1. We found a substantial reduction in the number and size of DIPG13 colonies in the presence of IGF-Trap (Figure 5E,G).

#### 2.5.3. The IGF-Trap Increases pHGG Apoptosis

To test the ability of IGF-1 to rescue the pHGG cells from apoptosis, the cells were maintained in a medium depleted of growth factors (DIPG13) or serum (SJG2) in the presence or absence of IGF-1. The incorporation of a fluorescently labelled Annexin V reagent by these cells was measured in real time using the Incucyte system. IGF-1 rescued DIPG13 from starvation-induced apoptosis, and this could be reversed by the IGF-Trap (Figure 5G,H). Exogenous IGF-1 had a minor rescue effect on SJG2 cells, but the addition of IGF-Trap, nevertheless, increased the apoptotic index for these cells (Figure 5I).

### 2.6. IGF-1 Increases Cell Cycle Progression in DIPG13 Cells, and This Is Blocked by the IGF-Trap

**Figure 6:**
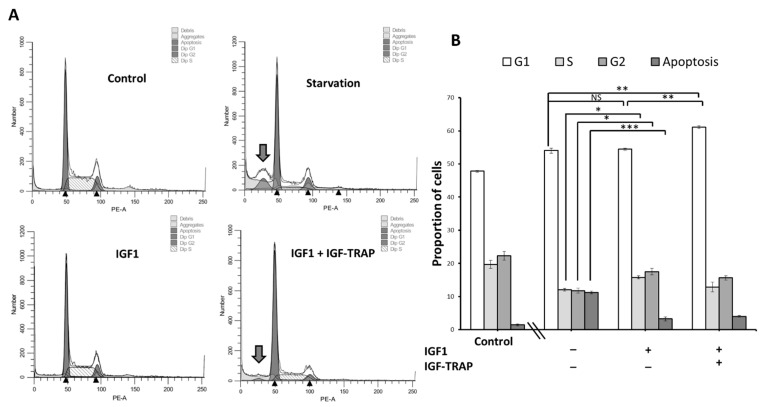
**The IGF-Trap blocks IGF-1-induced G_1_–S cell cycle transition.** To analyze cell cycle progression, DIPG13 cells were starved in minimal DMEM/F12 medium for 18 h and then incubated for 24 h in complete medium or in DMEM/F12 containing 50 ng/mL of IGF-1. The cells were fixed and stained with proprium iodide, and cell cycle analysis was performed by flow cytometry. Shown are representative flow cytometry histograms (**A**) where an arrow denotes apoptosis. Shown in the bar graphs (**B**) are the calculated proportions of cells at different cell cycle phases, including the proportions of apoptotic cells. Results are based on three independent experiments and are expressed as mean percentages (±SE) of total cells analyzed**.** * *p* < 0.05, ** *p* < 0.01, *** *p* < 0.001. NS—not significant.

Finally, having established that IGF-1R activated cyclin D1 transcription in the DIPG13 cells, we asked whether this affected the cell cycle progression in these cells. Cells were cultured in medium depleted of all growth supplements and treated with IGF-1 (or supplements, as positive control) for 24 h in the presence or absence of IGF-Trap, and the cell cycle analysis was performed by flow cytometry. As expected, we found that starvation halted cell cycle progression in these cells and induced apoptosis. The addition of IGF-1 enhanced G_1_-S transition, restoring it to levels similar to those observed in complete medium, and this was reversed by the addition of the IGF-Trap (Figure 6A,B), confirming the role of IGF-1 as a driver of cell cycle progression in these cells.

## 3. Discussion

The aims of this study were to evaluate IGF-responsiveness in human pediatric high-grade glioma cells and to assess the sensitivity of the cells to a novel IGF-inhibitor, the IGF-Trap. We selected for the study two pHGG PDXs with distinct genomic perturbations representing different clinical subtypes, and we confirmed their IGF-1R expression and responsiveness. In addition, we confirmed nuclear IGF-1R localization in several pHGG diffuse midline PDXs, consistent with findings by Clément et al that identified nuclear IGF-1R localization as a signature of pediatric high-grade gliomas [4].

IGF signaling is known to trigger the MEK/ERK and PI3K/Akt pathways that result in increased cell proliferation and survival, respectively. We confirmed that in response to IGF-1, the receptor was activated in both DIPG13 and SJG2 cells and showed that the proliferation of DIPG13 cells increased; they could also be rescued from starvation-induced apoptosis in the presence of IGF-1. Because patient-derived xenografts are cultured in defined media with low or no serum to maintain stemness, the optimal conditions for the IGF-1-mediated effects had to first be established, and were found to be cell type-specific. Under these conditions, we did not find a significant increase in SJG2 proliferation in response to IGF-1. Intriguingly, however, the proliferation of both DIPG13 and SJG2 cells was inhibited by the IGF-Trap. This is likely due to the higher expression of IGF-1 in the SJG2 cells that could drive autocrine IGF-1R signaling, even in the absence of exogenous IGF-1.

In both DIPG13 and SJG2 cells, IGF-1 stimulation resulted in the nuclear translocation of the receptor, and this upregulated IGF-1R and cyclin D1 expression in DIPG13 cells independently of MEK/ERK activation and resulted in enhanced G_1_-S transition in the minimal medium supplemented with IGF-1 only. We did not, however, observe cyclin D1 upregulation in the SJG2 cells treated with IGF-1, suggesting that the cellular context determines the transcriptional activity of the nuclear IGF-1R. Of relevance, recent results of a meta-analysis of over 1000 pediatric high-grade gliomas and DIPG cases identified amplifications in cell cycle genes including CCND1, CDK4 and CDK6 among the genetic aberrations in subclonal populations of DIPG [26,27], suggesting that cell cycle drivers may be potential targets in this incurable disease [27]. Hence, combining IGF-axis and CDK4/6 inhibitors may hold promise for DIPG treatment [26].

The standard of care for pediatric brain tumors is currently a combination of surgery, chemotherapy and radiation therapy—modalities that are associated with severe toxicity and have had limited curative effect. Although there is only scant information on the effect of IGF axis targeting in pHGG, several reports suggest that a systematic analysis of the beneficial effects of IGF-inhibitors is warranted. Thus, Bielen et al., using pediatric glioblastoma (pGBM) cell lines, showed that the specific IGF-1R small molecule inhibitor NVPAEW541 or receptor silencing by siRNA decreased cell viability and induced G1 arrest in the cells, and that co-treatment of the cells with the PDGFR inhibitor imatinib and NVP-AEW541 resulted in a highly synergistic interaction in vitro. This combination therapy also reduced the growth of the pGBM in vivo [28], identifying IGF-1R as a potential target in this disease and suggesting that combinatorial therapy with other RTK inhibitors may optimize the response. More recently, Simpson et al. showed that IGF-1R targeting increased the radio-sensitivity of pHGG cells, likely through perturbation of the DNA damage response [14]. Of relevance, IGF-1R was also identified as a therapeutic target in medulloblastoma, a highly aggressive pediatric malignancy of the cerebellum [29,30]. Collectively, the results suggest that IGF-1R targeting may have beneficial therapeutic effects in pediatric brain malignancies, including pHGG. IGF-targeting drugs are generally well tolerated, and to date, no deleterious effects have been observed in animals treated with the IGF-Trap [9,10]. Our results warrant further investigation of the sensitivity of pHGG PDX implanted orthotopically in the brain to IGF-Trap treatment. However, obstacles such as the very slow growth rate of pHGG PDX cells in the brain and the poor access of biologics such as the IGF-Trap to the tumor site due to the blood-brain barrier will need to be overcome to achieve a therapeutic effect in vivo.

## 4. Materials and Methods

Cells: The patient-derived primary tumor cell lines (DIPG13 BT245, HSJ19 and HSJ51) were described in detail previously [16]. DIPG13 and BT245 were kind gifts from Dr. Michelle Monje (Stanford University, San Francisco, CA, USA) and Dr. Keith Ligon (Dana-Farber Cancer Institute, Boston, MA, USA); they were received in September 2014 and November 2015, respectively. HSJ-019 and HSJ-051 resections were obtained in September 2015 and July 2016, respectively, from the Department of Neurosurgery of St. Justine Hospital (Montreal QC, Canada), and PDX lines were developed at the Research Institute of the McGill University Health Center (MUHC RI). These cells were all authenticated using the Microsatellite Geneprint 10 analysis (Genome, Montreal, QC, Canada) upon reception and periodically after 4 (HSJ-19), 7 (HSJ-51), or 9 (DIPG13, BT245) passages. *To preserve stemness*, *the cells were plated on laminin-coated culture dishes and maintained in Neurocult NSC proliferation media* (STEMCELL Technologies—Vancouver, BC, Canada) containing 0.0002% heparin and supplemented with 10 ng/mL basic fibroblast growth factor (bFGF, Wisent-St. Bruno, QC, Canada) and 20 ng/mL epidermal growth factor (EGF, Peprotech—Cranbury, NJ, USA) (Sigma, Oakville, ON, Canada). The tumor-derived cell lines were confirmed to match original samples by STR fingerprinting [16]. The SJ-GBM2 cells (also known as SJG2, the designation used throughout this manuscript) are part of the NCI Pediatric Preclinical Testing Program (PPTP) cell line panel. These cells were maintained in DMEM/F12 medium (Wisent—St. Bruno, QC, Canada) with 10% fetal bovine serum (FBS). The STR electropherograms for all lines are shown in Supplementary Figure S3. All Cells were maintained as a frozen stock and cultured briefly prior to use in the experiments throughout this study.

Reagents and Antibodies: Recombinant human IGF-1 was from R&D Systems (Toronto, ON, Canada). Rabbit monoclonal anti-IGF-1R (ab182408) and p-IGF-1R (Y-1161; ab39398) antibodies were from Abcam (Cambridge, MA, USA). Antibodies to ERK (9102S), p-ERK (9101S), GAPDH (2118S) Akt (9272S) p-Akt (3787S) and Cyclin D1 were all from Cell Signaling (Beverly, MA, USA). Antibody to tubulin (T9028) was from Sigma. Secondary antibodies Alexa Fluor 488-goat-anti-rabbit was from Life Technologies (Burlington, ON, Canada) and DAPI from Invitrogen (Burlington, ON, Canada). The MEK inhibitor (PD98059) was from Calbiochem (Sigma-Aldrich Canada, Oakville, ON, Canada).

IGF-1R activation and signaling: Cells were cultured in complete medium for 24 h, followed by starvation media (DMEM/F12 medium) for 16 h, prior to stimulation with 50 (DIPG13) or 10 (SJG2) ng/mL of IGF-1 in the presence or absence of IGF-Trap (2:1 IGF-Trap:IGF-1 molar ratio). Following stimulation, the cells were placed on ice, rinsed twice with ice cold PBS, and lysed directly on the plate with the RIPA lysis buffer (50 mM Tris (pH 7.4), 1 mM EDTA, 150 mM NaCl, 1% (*w*/*v*) NP-40, 2 mM Na_3_VO_4_, 5 mM NaF, 0.25% sodium deoxycholate and a protease inhibitor cocktail (Roche, Mississauga, ON, Canada)). The lysates were transferred to 1.5 mL tubes, vortexed for 30 s, and incubated on ice for 5 min. Tubes were centrifuged at 13,000× *g* for 10 min at 4 °C, and supernatants were transferred to clean 1.5 mL tubes. Cell lysates were quantified using the BCA assay and analyzed by Western blotting.

RNA extraction and qPCR: Cells were cultured in complete media (DIPG13) or in DMEM/F12 containing 1% FBS (SJG2) for 48 h. Total cellular RNA was extracted using Trizol (Life Technologies, Burlington, ON, Canada), and qPCR was performed using standard procedures as we previously described [31]; the primers were described in Supplementary Table S1, and the FASTSTART Universal SYBR Green reagent (Roche Pharmaceuticals, Bedford, MA, USA) were used according to manufacturer’s instructions. The Applied Biosystems 7500 Fast real-time cycler was used to analyze the samples.

Immunocytochemistry (ICC): Cells were seeded on laminin-coated coverslips (2 × 10^4^ cells/coverslip), incubated for 48 h at 37 °C, fixed for 20 min at room temperature in 3.7% paraformaldehyde in 5% sucrose, and washed repeatedly in PBS. Cells were permeabilized in 0.1 or 0.2% Triton X-100. The coverslips were incubated in blocking medium (5% serum in PBS) followed by incubation for 1 h at room temperature with the indicated primary antibodies and a 30 min incubation at room temperature with the appropriate Alexa Fluor-conjugated secondary antibody. The coverslips were counterstained with DAPI and mounted in ProLong Gold mounting reagent. Images were analyzed using a Zeiss LSM780 laser scanning confocal microscope (Zeiss, Jena, Germany) equipped with an AxioCam camera.

Subcellular fractionation: Cells were cultured for 18 h in basal media (DMEM/F12) depleted of growth supplements and growth factors, and then stimulated with 10 or 50 ng/mL of IGF-1 for the duration indicated. The cells were lysed on ice with a hypotonic buffer (20 mM HEPES, 1 mM EDTA, 1 mM EGTA, 1 mM DTT, protease inhibitors, and phosphatase inhibitors) containing 0.2% NP40. The lysates were centrifuged at 4 °C for 20 s at 15,000 rpm; the supernatants containing the cytoplasmic fractions were collected, and the pellets containing nuclei were washed twice with hypotonic buffer and resuspended in a high salt buffer (420 mM NaCl, 20 mM HEPES, 1 mM EDTA, 1 mM EGTA, 20% glycerol, 1 mM DTT, 0.5 mM PMSF) containing protease and phosphatase inhibitor cocktails and incubated on a rotating shaker for 30 min at 4 °C. Lysates were centrifuged for 20 min at 15,000 rpm, and the supernatants containing the nuclear fractions were collected. Both fractions were stored at −80 °C until used.

Immunoblotting: Immunoblotting was performed as we described in detail previously [32]. Briefly, cells were lysed in RIPA lysis buffer. Following centrifugation at 13,000× *g* for 10 min, lysates were separated by SDS-PAGE using 8 or 10% polyacrylamide gels and transferred onto polyvinylidene difluoride (PVDF) or nitrocellulose membranes. Membranes were blocked in 5% BSA in TBST (Tris buffered saline with 0.1% Tween) for 1 h, followed by incubation, first at 4 °C overnight with the primary antibody, and then for 1 h at room temperature with HRP-conjugated anti-rabbit or anti-mouse immunoglobulin secondary antibodies (Jackson ImmunoResearch—West Grove, PA, USA), as appropriate. Signal detection and densitometry were performed using ImageQuant Las4000.

Analysis of cell proliferation in real time: DIPG13 and SJG2 cells (3 × 10^4^ cells/well) were seeded in 48- and 24-well plates, respectively, and incubated in complete medium at 37 °C overnight. Prior to analysis, the cells were maintained in culture medium depleted of growth factors or specific supplements as indicated, and they were supplemented with 10 (SJG2) or 50 (DIPG13) ng/mL or IGF-1. The cells were placed in the Incucyte live cell imaging system (Essen Bioscience, Ann Arbor, MI, USA), and images were acquired every 6 h for 4 days and analyzed using the phase confluence Incucyte setting.

MTT Assay: SJG2 cells were seeded in 96-well plates at a density of 10^4^ cells/well and incubated overnight at 37 °C in complete medium. Cells were washed and cultured in growth-supplement-depleted medium as indicated, in the presence or absence of 10 ng/mL IGF-1 and with or without the indicated concentrations of the IGF-Trap. The MTT reagent was added and incubated for 3–4 h at 37 °C; the formazan crystals were dissolved in DMSO, and absorbance was recorded at 570 nm.

Apoptosis Assay: Cells were seeded in 96-well plates and incubated overnight at 37 °C in complete medium. The medium was then replaced with growth-supplement-depleted medium as indicated, and the cells were incubated in the presence or absence of IGF-1 and with or without the indicated concentrations of IGF-Trap. The Annexin V red reagent (Essen Bioscience, Ann Arbor, MI, USA) was added, and cells were analyzed in the Incucyte live cell imaging system. Images were acquired every 3 h for 5 days, and Incucyte Integrated Analysis software was used to analyze the data.

Cloning assay: DIPG13 (500 cells/well) were seeded onto laminin-coated 6-well plates and cultured in complete medium with or without 50 ng/mL of IGF-1 and in the presence or absence of IGF-Trap (1:1 molar ratio to IGF-1). The medium was replenished every 4 days for up to 15 days, at which time colonies were fixed in 4% paraformaldehyde and stained with 0.5% crystal violet. Colonies were counted and sized using a Nikon Eclipes Ts2 microscope and NIS-Elements software.

Cell Cycle analysis: Cell cycle was analyzed by flow cytometry. Cells in 6-well plates (5 × 10^4^ cells/well) were starved (in DMEM/F12) overnight for 18 h before treatment for 24 h with the indicated concentrations of IGF-1, IGF-Trap or both, vehicle (PBS), or complete medium (as positive control). Cells were collected, centrifuged at 1000 rpm for 5 min, washed twice with PBS, and fixed for 20 min in chilled 70% ethanol. Cells were stained with propidium iodide (20 μg/mL), treated with RNase A (100 μg/mL) for 2 h at 4 °C in the dark, and debris were removed using a cell strainer (100 μm) before the analysis. Cell cycle phases were determined by acquiring at least 10^4^ events using the FACSCanto (BD Biosciences, San Jose, CA, USA) and the data were analyzed using the ModFit software (Verity Software House, Topsham, ME, USA).

Statistical Analysis: The experiments were performed three times each in triplicate, and the Student’s *t*-test was used for all statistical analyses.

## 5. Conclusions and Significance

Pediatric high-grade glioma (pHGG) has a dismal prognosis. Studies on pHGG biology have been hampered by a lack of clinically-relevant models. Here, we used patient-derived pHGG cells that were maintained under conditions of stemness to investigate IGF-1 receptor signaling and targeting in pHGG cells. We showed that the IGF-1 receptor promotes pHGG growth via canonical ERK and PI3K/Akt signaling and by translocating to the nucleus to activate cyclin D1 transcription. We found that both pathways could be inhibited by the IGF-Trap, a novel IGF-1R inhibitor, resulting in reduced cell proliferation and colony formation, increased apoptosis, and an inhibition of cell cycle progression. The results identify the IGF-axis as a potential target in pediatric high-grade glioma.

## Data Availability

Data is contained within the article and Supplementary Materials.

## Supplementary Materials

The following supporting information can be downloaded at: https://www.mdpi.com/article/10.3390/ph16020297/s1, Figure S1: The IGF-Trap does not alter signaling in SJG2 cells. Figure S2: IGF-1 does not regulate Cyclin D1 transcription in SJG2 cells. Figure S3: Shown are STR electropherograms of all patient-derived pediatric high grade glioma lines used in this study. Dates of the analyses are indicated in the individual titles. Table S1: List of primer sets used to analyze RNA samples by qPCR.

## Abbreviations

IGF-1: Type 1 insulin-like growth factor; IGF-1R: IGF-1 receptor; pHGG: pediatric high-grade gliomas; DIPG: diffuse intrinsic pontine glioma; SUMO-1: small ubiquitin-like modifier protein–1; AKT: Serine threonine kinase 1, also known as protein kinase B (PKB); ERK: extracellular signal-regulated kinase; MEK: mitogen activated protein kinase kinase.
