# Supplementary material for: Targeting the IGF-Axis in Cultured Pediatric High-Grade Glioma Cells Inhibits Cell Cycle Progression and Survival
Source: Pharmaceuticals (Basel). 2023 Feb 14;16(2):297. doi: 10.3390/ph16020297 (PMC9964639; doi:10.3390/ph16020297)
Supplement: Supplementary file 1 [file pharmaceuticals-16-00297-s001.zip › pharmaceuticals-2169233-supplementary.pdf]

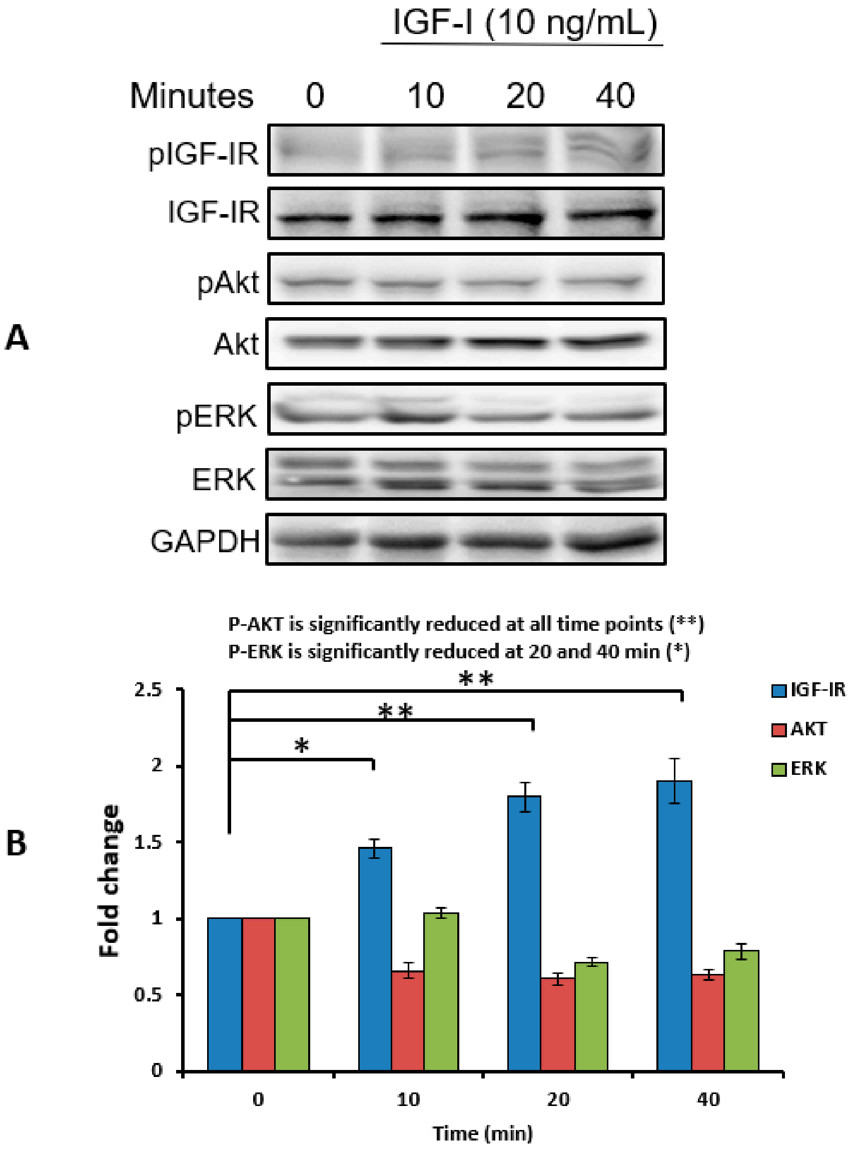

Figure S1. The IGF-Trap does not alter signaling in SJG2 cells. (**A**) Shown on top are representative results of Western blotting performed following stimulation of serum starved SJG2 cells with 10 ng/mL IGF-1. (**B**) Shown in the bar graphs (bottom) are the means and SE based on 3 experiments. * $p < 0.05$, ** $p < 0.01$.

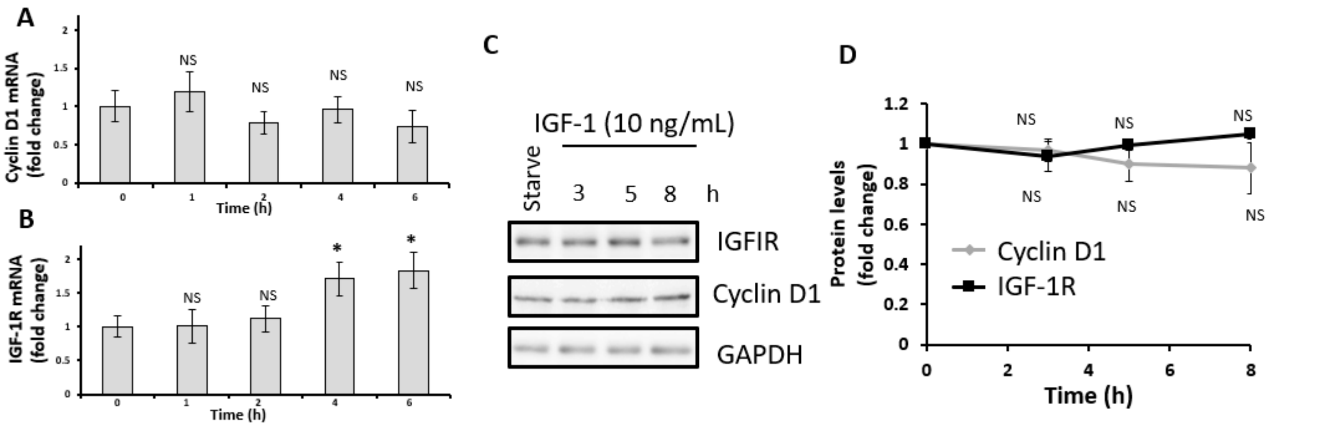

Figure S2. IGF-1 does not regulate Cyclin D1 transcription in SJG2 cells**.** Shown in (**A**,**B**) are qPCR results obtained for Cyclin D1 (**A**) and IGF-1R (**B**) transcripts and in (**C**,**D**) results of Western blotting performed on lysates of cells stimulated with 10 ng/mL IGF-1. Results of densitometry are expressed as fold change in the indicated protein levels relative to levels at time 0 that were assigned a value of 1 (n = 3). * $p \leq 0.05$, NS—Not significant.

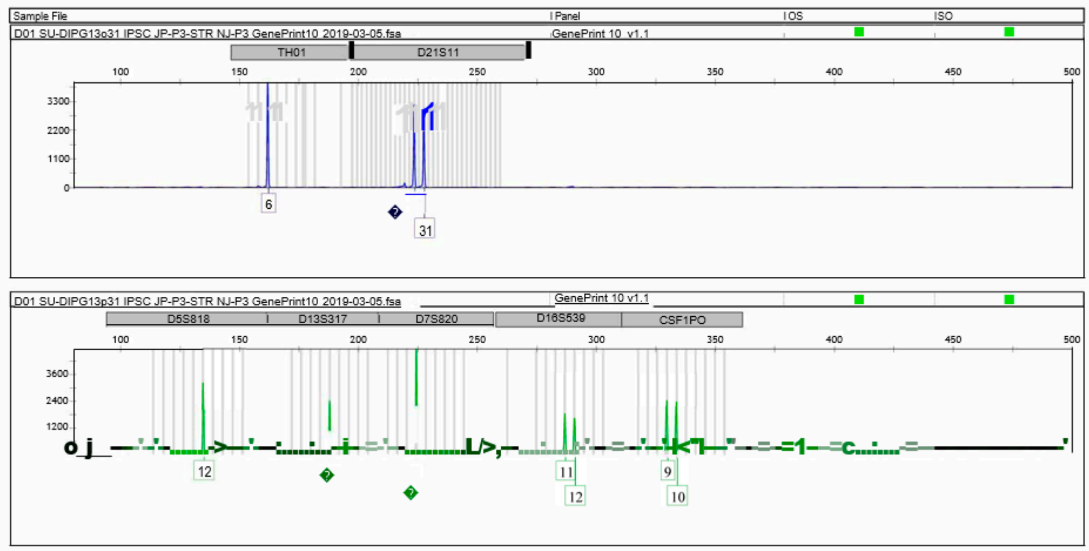

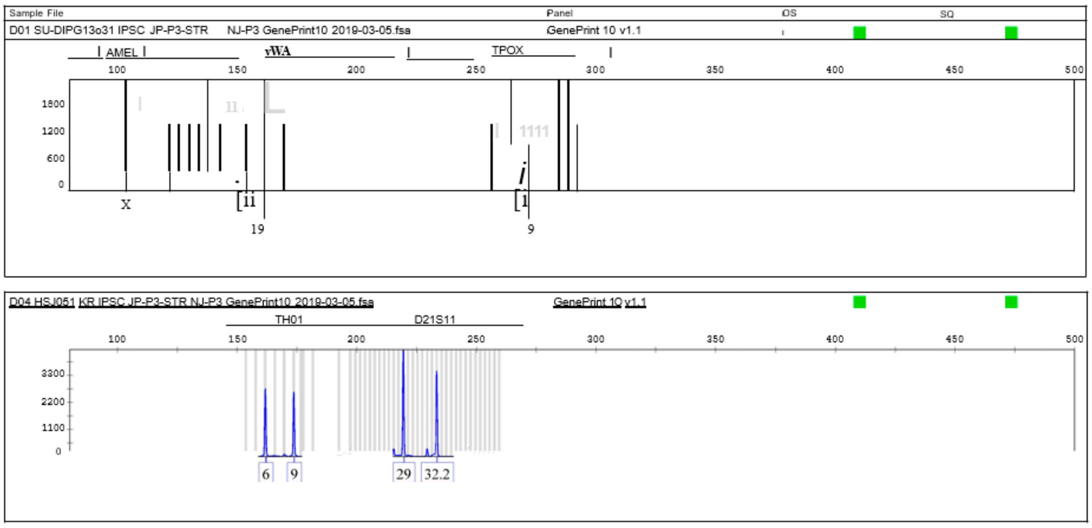

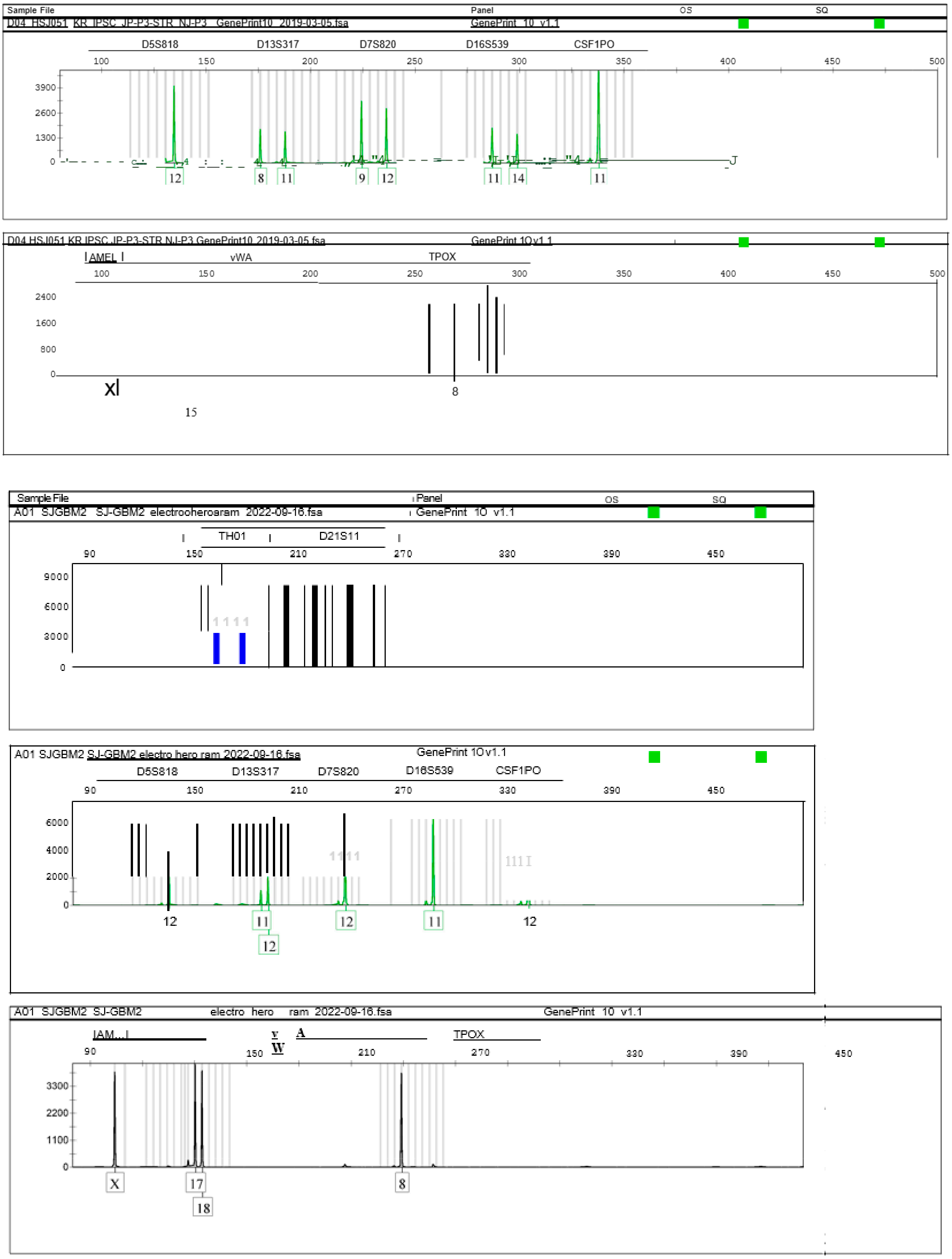
Sample File
D04 HSJ051 KR IPSC JP-P3-STR NJ-P3 GenePrint10 2019-03-05.fsa
Panel
GenePrint 10 v1.1
OS
SQ
D5S818
D13S317
D7S820
D16S539
CSF1PO
12
8
11
9
12
11
14
11
AMEL
vWA
TPOX
X
15
8
Sample File
A01 SJGBM2 SJ-GBM2 electroheroaram 2022-09-16.fsa
Panel
GenePrint 10 v1.1
TH01
D21S11
A01 SJGBM2 SJ-GBM2 electro hero ram 2022-09-16.fsa
GenePrint 10 v1.1
D5S818
D13S317
D7S820
D16S539
CSF1PO
12
11
12
12
11
12
A01 SJGBM2 SJ-GBM2 electro hero ram 2022-09-16.fsa
GenePrint 10 v1.1
TPOX
X
17
18
8

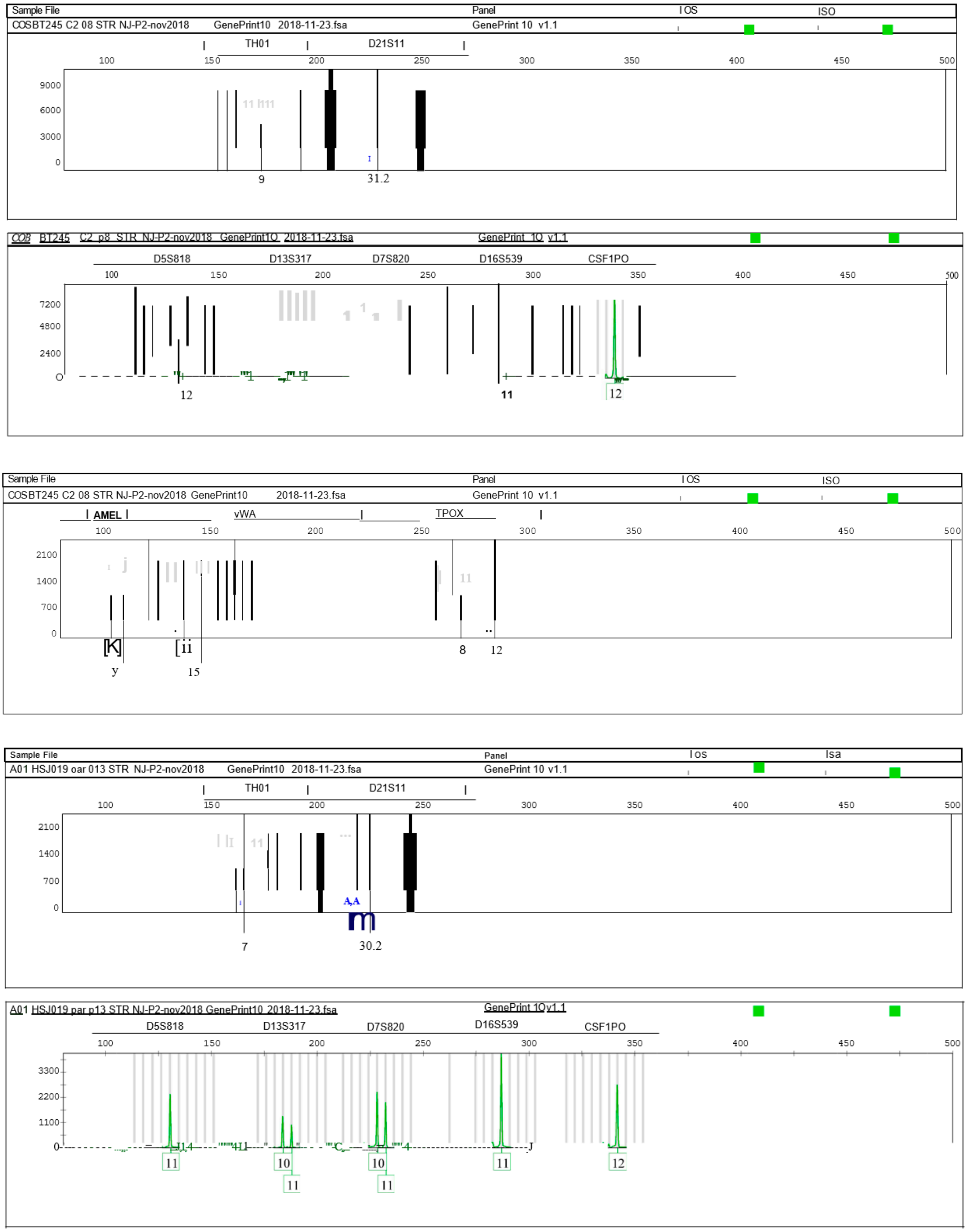
Sample File
COSBT245 C2 08 STR NJ-P2-nov2018 GenePrint10 2018-11-23.fsa
Panel
GenePrint 10 v1.1
TH01
D21S11
9
31.2
D5S818
D13S317
D7S820
D16S539
CSF1PO
12
11
12
AMEL
vWA
TPOX
Y
15
8
12
A01 HSJ019 oar 013 STR NJ-P2-nov2018 GenePrint10 2018-11-23.fsa
7
30.2
11
10
11
10
11
11
12

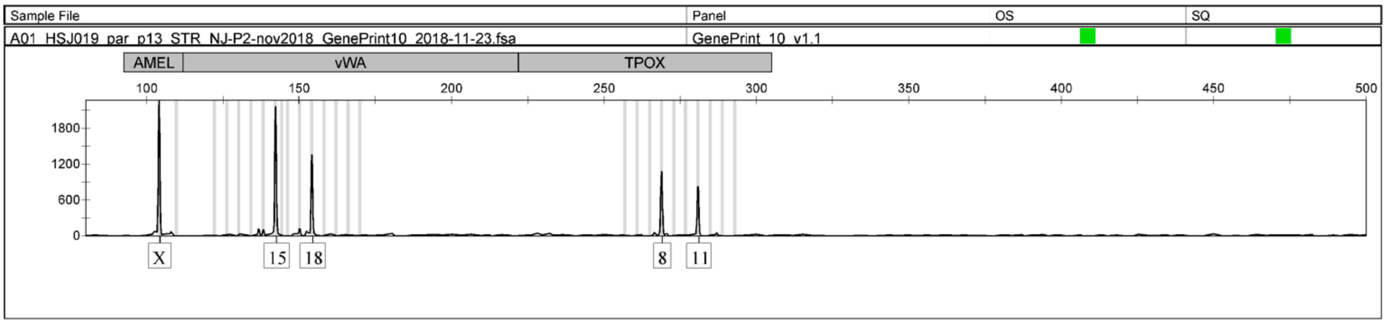

Figure S3. Shown are STR electropherograms of all patient-derived pediatric high grade glioma lines used in this study. Dates of the analyses are indicated in the individual titles.

Table S1. List of primer sets used to analyze RNA samples by qPCR.

Gene	Direction	Primer 5′→3′
GAPDH	Forward	GGATTTGGTCGTATTGGGCG
	Reverse	ATGGAATTTGCCATGGGTGG
IGF-1R	Forward	CGCACCAATGCTTCAGTTCC
	Reverse	TGCCAGCGCACAATGTAGTA
IGF-1	Forward	CTCTTCAGTTCGTGTGTGGA
	Reverse	CAGCCTCCTTAGATCACAGC
IGF-2	Forward	TGGCATCGTTGAGGAAGTGCT
	Reverse	ACGGGGTATCTGGGGAAGTT
Cyclin D1	Forward	TGAGGAGCCCCAACAACTTC
	Reverse	CCGGGTCACACTTGATCACT
